# Characteristics of pandemic influenza A (H1N1) infection in patients presenting to a university hospital in Riyadh, Saudi Arabia

**DOI:** 10.4103/0256-4947.59377

**Published:** 2010

**Authors:** Abdulaziz A. BinSaeed

**Affiliations:** **On behalf of the Standing Committee of Epidemic Control, College of Medicine and King Khalid University Hospital, King Saud University, Riyadh, Saudi Arabia**

## Abstract

**BACKGROUND AND OBJECTIVES:**

A national plan of management for flu-like illnesses was developed by the Saudi Ministry of Health after the first outbreak in Saudi Arabia in June. We describe the clinical presentation of the H1N1 cases attending King Khalid University Hospital (KKUH) between July through September 2009 and identify the high-risk age groups.

**METHODS:**

All patients presenting with influenza-like illnesses (ILI) in the H1N1 clinics during the specified period were clinically examined and tested using reverse transcription polymerase chain reaction (RT-PCR). Those who were clinically diagnosed and confirmed positive for novel influenza A (H1N1) were included in the study.

**RESULTS:**

Over a 6-week period, 117 cases of laboratory-confirmed cases were reported in KKUH with a mean (SD) age of 19.6 (16.7) years, of whom 72 (62.1%) were males. Most reported cases were Saudis (n=99, 85.3%); 94 (81%) had no travel history outside the country; 100 (86.2%) had had no contact with an H1N1-identified patient; 33% were aged 5-14 years and 28.4% were aged 15-29 years. The most commonly reported symptoms were fever in 99 (85.3%), cough in 9 (81%), runny nose (33.6%) and sore throat (21.3%). All 117 cases were confirmed positive using real time RT-PCR testing. Thirty-one cases (26%) were admitted and 22 of those (71%) recovered after receiving oseltamivir. Two deaths were attributed to the 2009 pandemic. One patient died of chronic pulmonary disease. The other cause of death was unknown.

**CONCLUSION:**

These findings indicate indigenous influenza A (H1N1) transmission, and confirm the urgent need for prevention strategies which specifically target children and young adults, who appear to have a higher risk of infection and hospitalization. Such measures include immunization, improved personal hygiene, and increased ventilation in habitations.

The World Health Organization (WHO) Global Influenza Surveillance Network (GISN) continues to monitor the global circulation of influenza viruses and seasonal viruses. From the beginning of the declared pandemic on April 19 to September 12, 2009 a total of 80 countries had reported cases. The total number of specimens reported positive for influenza viruses by WHO-designated National Influenza Centers laboratories was 110 969. Of these, 67 207 (60.6%) were pandemic H1N1 and 6054 (5.5%) were seasonal A (H1). Data from issued reports issued for the week September 6 to September 12, 2009 (a total of 20 countries) show that the total number of specimens reporting positive for influenza viruses by NIC laboratories was 5862. Of these, 4444 (75.8 %) were pandemic H1N1 and 155 (2.6 %) were seasonal A (H1). On average, the pandemic A (H1N1) accounted for 76% of all subtypes of influenza detected (76% in the northern hemisphere and 87% in the southern hemisphere).^1^

On June 3, 2009 the first case of pandemic influenza A (H1N1) virus was reported in Saudi Arabia.^2^ On September 12, 2009, the Saudi Ministry of Health issued a national plan of management for flu-like pandemics, specifically pandemic influenza A (H1N1) virus infections.^3^ Moreover, the Saudi Ministry of Interior published an action plan to manage the mass influx of travelers arriving for the Islamic pilgrimages of Ummrah and Hajj.^4^ The action plan recommended obligatory pre-travel vaccination against seasonal and/or H1N1 influenza, an awareness campaign for preventive procedures, a compulsory health assessment and follow-up, and for their own safety, urged high-risk groups (elderly, patients with chronic diseases, children and pregnant women) to avoid travelling. This report summarizes laboratory-confirmed cases identified during the period from July 1 to September 12, 2009 at King Khalid University Hospital, Riyadh, Saudi Arabia.

## METHODS

The data was collected using a specially designed form based upon KKUH medical records covering a period of 6 weeks (July 1 to September 12, 2009). A total of 117 subjects, identified through H1N1 clinics were diagnosed for influenza-like illness on a clinical basis and confirmed positive for novel influenza A (H1N1) through RT-PCR testing (Roche, Germany). Subjects who reported having an ILI (defined as an oral temperature of more than 38°C (100.4°F) or a history of fever or chills and at least one influenza-like symptom) were asked to provide specimens for virologic testing via nasal and throat swabs. The diagnostic test was a real-time RT-PCR assay that uses fluorogenic hydrolysis probe technology for the detection of human influenza A viruses, and the differential detection of 2009 H1N1 influenza virus in nasopharyngeal swabs (NPS), nasal swabs (NS), throat swabs (TS), and nasal aspirates (NA) according to the manufacturer's instructions (Roche, Germany) using specific probes for the novel influenza A (H1N1) strain.

## RESULTS

By September 12, 2009 a total of 117 laboratory confirmed cases were reported at KKUH. Of these, 72 (62.1%) were males and 42 (36.2%) females. Gender information was missing in three cases. The mean age was 19.6 (16.7) years. The age of cases ranged from 2 months to 75 years. Nevertheless, most reported cases were in the age range of 5 to 14 years followed by 15 to 29 years (Table 1). The fewest cases were reported for children aged 0 to 4 years. The majority of the patients (n=99, 85.3%) were Saudis. The remainder included 7 Filipinos, 1 Palestinian and 1 Pakistani. Nationality information was missing in nine cases. Most of these patients (n=94, 81%) had not travelled out of the country, while 14 had travelled within the Middle East, 6 to Europe and only 2 to Asia. Most interestingly, an unexpectedly large number of cases confirmed that they had not had contact with an H1N1-identified patient (n=100, 86.2%) nor any contact with a patient with any symptoms of influenza (n=83, 71.6%).

**Table 1:** Laboratory-confirmed H1N1 cases reported to King Khalid University Hospital, July 1 –September 12, 2009, by age.

Age groups in years	No. of H1N1 cases	Percent
0-4	18	16.5
5-14	36	33.0
15-29	31	28.4
30-59	21	19.3
≥60	3	2.8
**Total**	**109**	**100**

Information missing in 8 patients

Among 116 patients for whom the data for signs and symptoms were available, the most commonly reported symptoms are shown in Table 2. The most frequently reported groups of symptoms were: fever and cough (n=30, 25.9%); fever, cough and sore throat (n=19 patients, 16.4%); and fever, cough and runny nose (n=13, 11.2 %).

**Figure 1:**
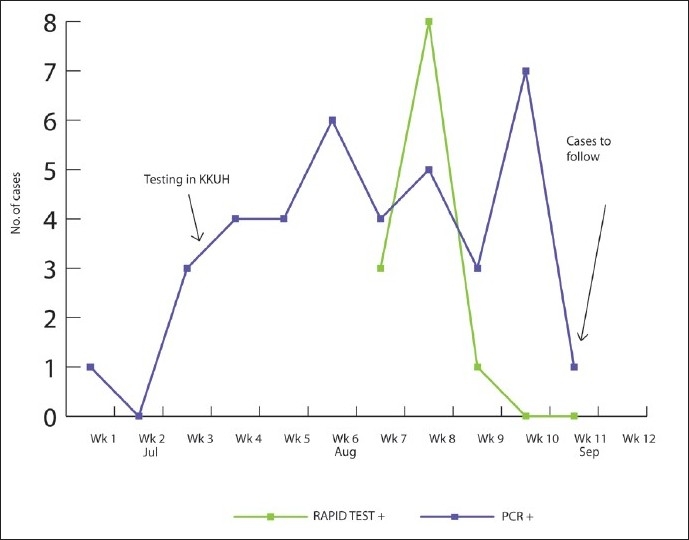
Laboratory-confirmed cases of H1N1 reported to King Khalid University Hospital, July 1 to September 12, 2009.

Figure 1 shows the number of cases that were confirmed positive using the Rapid Test (Becton-Dickinson, USA) and the polymerase chain reaction (PCR) test over 12 weeks from July 1 to September 12, 2009. Of the total number of cases, only 25 were tested for H1N1, with the Rapid Test showing 15 cases as positive (11 reported in the month of August and 4 in September), while the remaining 10 cases were reported negative. As per the selection criteria, all 117 cases were confirmed positive using the PCR test. According to the results, 15 cases were reported in July, 55 in August and 45 in September. Two cases had missing data on the date of diagnosis. Prior to July 15, 2009, KKUH referred the screening Rapid Test and PCR for H1N1 to external diagnostic laboratories. The start date of testing within KKUH laboratories is marked on Figure 1. For the data recorded as of week 12 (mid-week of September 2009), most cases, (n=85, 73%) were diagnosed in the outpatient clinic and sent home with specific medical advice. About one-third (31 cases) were admitted as in-patients, out of whom 22 recovered after receiving a treatment regimen of oseltamivir phosphate (Tamiflu, Roche) capsules for adults and a suspension for children in a dose of 75 mg twice per day for five days. All patients were subsequently discharged. Nine inpatients were still undergoing treatment when this report was prepared. The data also showed that out of those admitted, 12 cases suffered from co-morbidities (2 with pulmonary disease; 1 with cardiac disease; 2 with chronic renal/liver diseases; 1 with diabetes; 1 was overweight as determined by BMI; and 5 were suffering other diseases). Additionally, there was one pregnant woman and a lactating mother with a reported decreased milk volume. As of September 12, 2009 there were two reported deaths of west Riyadh residents attributed to the 2009 pandemic influenza A (H1N1) virus infection; one had chronic pulmonary disease and the other, from outside the KKUH, had unknown contributory diseases.^5^

**Table 2:** Laboratory -confirmed H1N1 cases reported to King Khalid University Hospital, July 1 to September 12, 2009, by signs and symptoms.

Multiple symptoms reported	No. of H1N1 cases	Percentage (n=117)
Adult symptoms		
Shortness of breath	17	14.7
Fever for more than 3 days while being medicated	7	6
Chest pain	6	5.2
Hemoptysis	3	2.6
Cyanosis	2	1.7

Pediatric symptoms		
Shortness of breath and tachypnea	19	16.4
Disinterest in playing	3	2.6

Common signs and symptoms		
Fever	99	85.3
Cough	94	81
Running nose	39	33.6
Sore throat	25	21.3
Vomiting	11	9.5
Body pain	10	8.5
Headache	8	6.8
Unspecified	6	5.1
Rigor	5	4.3
Diarrhea	4	3.4
Chills	3	2.6
Eyelid puffiness	2	1.7
Nausea	2	1.7
Epigastic pain	1	0.8
Body weakness	1	0.8
Fatigue	1	0.8
Abdominal pain	1	0.8
Decreased feeding	1	0.8

## DISCUSSION

To raise awareness about the status of the novel influenza A (H1N1) and prevention and control efforts, the King Saud University (KSU) of Riyadh established the Standing Epidemic Control Committee (SECC). The SECC has sent several health educational alerts to KSU employees and students, as well as the nearby general community through the University media and electronic network. It is thought that these messages contributed to the successful early identification of cases. Thus, as of September 12, 2009 the 117 cases reported to KKUH (one of about 100 public and private hospitals in Riyadh) constituted 6% of the overall nationally reported confirmed cases at that time. This reflects the high awareness of KKUH health-care providers (including different health professionals) of the outbreak, leading to high public awareness, which could have enhanced hospital laboratory surveillance activities.

During the 6-week period covered by this report, the 117 confirmed cases of 2009 pandemic influenza A (H1N1) virus infection were reported to the Infection Control Unit at KKUH by the H1N1 university clinics. The highest proportion of cases, both overall and among hospitalized patients, were among children aged 5 to 14 years or adolescent/young adults aged 15 to 29 years (>60%), with a substantially lower proportion aged ≥60 years. Recent reports from Mexico and USA^6^,^7^ indicating that age-specific attack rates for the 2009 pandemic Influenza A (H1N1) virus infection cases are higher in younger persons and lower in older persons (compared with seasonal influenza infections) concur with the results of this study. Further explanations are provided by two Center for Disease Control (CDC, US) reports, which suggest that older persons, as a group, may have pre-existing immunity to the 2009 H1N1 virus^8^ or a cross-reactive antibody to it, compared with none detected among children.^9^ Another factor which may contribute to higher rates among children/adolescents could be greater contact rates among teenagers, as reported by a recent Japanese study.^10^ Such an age differential could be also be attributed to a variation in exposure and susceptibility in accordance with the transition from a younger to older age.

The great majority of affected patients reported that they had not travelled outside of the country (81 %), had not had contact with any H1N1-identified patient (86.2%) and had not had contact with a patient with symptoms of influenza (71.6%). Such findings suggest an indigenous transmission of the novel influenza A (H1N1) virus within the country, which may have significant implications in rapid spread of the infection during mass gatherings. These findings also confirm the urgent need for prevention strategies that specifically target children and young adults who are at a higher risk of infection and hospitalization. The national plan for management of influenza pandemics and the CDC Advisory Committee on Immunization Practices (ACIP)^11^ recommend that these population sub-groups should be among the first groups targeted for vaccination with the influenza A (H1N1) 2009 monovalent vaccine, once available and locally approved. The WHO mentions that the length of the approval process depends on factors such as each country's regulatory pathway, the type of vaccine being licensed, and the stage of manufacturers' readiness to submit appropriate information to regulatory authorities.^5^ The finding of three elderly cases above the age of 60 years in this study series negates a common medical myth in Saudi Arabia that a majority of the high-risk population has been vaccinated. Thus, it is recommended that the campaign of vaccination for high-risk groups in the population be mobilized efficiently. Moreover, the data on pregnant and lactating mothers serves as a potential empirical gap^12^ especially in a cultural context and implies the need for specific attention.
